# Deciphering Biosignatures in Planetary Contexts

**DOI:** 10.1089/ast.2018.1903

**Published:** 2019-08-22

**Authors:** Marjorie A. Chan, Nancy W. Hinman, Sally L. Potter-McIntyre, Keith E. Schubert, Richard J. Gillams, Stanley M. Awramik, Penelope J. Boston, Dina M. Bower, David J. Des Marais, Jack D. Farmer, Tony Z. Jia, Penelope L. King, Robert M. Hazen, Richard J. Léveillé, Dominic Papineau, Kaitlin R. Rempfert, Mónica Sánchez-Román, John R. Spear, Gordon Southam, Jennifer C. Stern, Henderson James Cleaves

**Affiliations:** ^1^ Department of Geology & Geophysics, University of Utah, Salt Lake City, Utah.; ^2^ Department of Geosciences, University of Montana, Missoula, Montana.; ^3^ Geology Department, Southern Illinois University, Carbondale, Illinois.; ^4^ Department of Electrical and Computer Engineering, Baylor University, Waco, Texas.; ^5^ Earth-Life Science Institute, Tokyo Institute of Technology, Tokyo, Japan.; ^6^ Electronics and Computer Science, Institute for Life Sciences, University of Southampton, Southampton, United Kingdom.; ^7^ Department of Earth Science, University of California, Santa Barbara, Santa Barbara, California.; ^8^ NASA Astrobiology Institute, NASA Ames Research Center, Moffett Field, California.; ^9^ Department of Astronomy, University of Maryland College Park (CRESST), College Park, Maryland.; ^10^ NASA Goddard Space Flight Center, Greenbelt, Maryland.; ^11^ Exobiology Branch, NASA Ames Research Center, Moffett Field, Californnia.; ^12^ School of Earth and Space Exploration, Arizona State University, Tempe, Arizona.; ^13^ Research School of Earth Sciences, The Australian National University, Canberra, Australia.; ^14^ Geophysical Laboratory, Carnegie Institution for Science, Washington, District of Columbia.; ^15^ Department of Earth and Planetary Sciences, McGill University, Montreal, Canada.; ^16^ Geosciences Department, John Abbott College, Sainte-Anne-de-Bellevue, Canada.; ^17^ London Centre for Nanotechnology, University College London, London, United Kingdom.; ^18^ Department of Earth Sciences, University College London, London, United Kingdom.; ^19^ Centre for Planetary Sciences, University College London, London, United Kingdom.; ^20^ BioGeology and Environmental Geology State Key Laboratory, School of Earth Sciences, China University of Geosciences, Wuhan, China.; ^21^ Department of Geological Sciences, University of Colorado Boulder, Boulder, Colorado.; ^22^ Earth Sciences Department, Vrije Universiteit Amsterdam, Amsterdam, The Netherlands.; ^23^ Department of Civil and Environmental Engineering, Colorado School of Mines, Golden, Colorado.; ^24^ School of Earth and Environmental Sciences, The University of Queensland, St. Lucia, Queensland, Australia.; ^25^ Program in Interdisciplinary Studies, Institute for Advanced Study, Princeton, New Jersey.

**Keywords:** Astrobiology, Biosignatures, Taphonomy, Extraterrestrial life, Extremophile.

## Abstract

Microbial life permeates Earth's critical zone and has likely inhabited nearly all our planet's surface and near subsurface since before the beginning of the sedimentary rock record. Given the vast time that Earth has been teeming with life, do astrobiologists truly understand what geological features untouched by biological processes would look like? In the search for extraterrestrial life in the Universe, it is critical to determine what constitutes a biosignature across multiple scales, and how this compares with “abiosignatures” formed by nonliving processes. Developing standards for abiotic and biotic characteristics would provide quantitative metrics for comparison across different data types and observational time frames. The evidence for life detection falls into three categories of biosignatures: (1) substances, such as elemental abundances, isotopes, molecules, allotropes, enantiomers, minerals, and their associated properties; (2) objects that are physical features such as mats, fossils including trace-fossils and microbialites (stromatolites), and concretions; and (3) patterns, such as physical three-dimensional or conceptual *n*-dimensional relationships of physical or chemical phenomena, including patterns of intermolecular abundances of organic homologues, and patterns of stable isotopic abundances between and within compounds. Five key challenges that warrant future exploration by the astrobiology community include the following: (1) examining phenomena at the “right” spatial scales because biosignatures may elude us if not examined with the appropriate instrumentation or modeling approach at that specific scale; (2) identifying the precise context across multiple spatial and temporal scales to understand how tangible biosignatures may or may not be preserved; (3) increasing capability to mine big data sets to reveal relationships, for example, how Earth's mineral diversity may have evolved in conjunction with life; (4) leveraging cyberinfrastructure for data management of biosignature types, characteristics, and classifications; and (5) using three-dimensional to *n*-D representations of biotic and abiotic models overlain on multiple overlapping spatial and temporal relationships to provide new insights.

## 1. Introduction

The search for extraterrestrial life is fundamentally referenced to Earth as the only known and accessible benchmark for comparison (a sample size of *n* = 1 problem), from the microscopic level up to the scale of our planet and its atmosphere, where life has perturbed planetary environments over long timescales (Judson, 2017). “Life” is a complex phenomenon, and here we refer to it as we know it today—a self-organized, self-replicating, and metabolically active molecular system that is carbon based (Pace, 2001). All of Earth's surface and subsurface waters have likely been in contact with microbes or their by-products since at least 3.5 Ga, when the first widely accepted traces of life appear in the geological record (*cf.*, Schopf *et al.*, 2018 and references therein). That traces of life are preserved within very ancient remnants of the crust indicates that perhaps life had already colonized the entire planet. Accordingly, over the eons during which water-rich Earth has been teeming with life, it is difficult to determine how an “uninhabited habitable planet” would appear when sampled directly or observed remotely.

It has often been generally assumed that substances or objects in Earth's near-surface environment might be abiotic unless there is definitive evidence of biological activity. However the pervasiveness of life in Earth's near-surface and subsurface environments indicates that, conversely, perhaps virtually everything might be biologically influenced unless an abiotic origin can be definitively established.

## 2. Biosignature Definitions

A biosignature is an object, substance, and/or pattern whose origin specifically requires a biological agent (Des Marais *et al*., 2008). The usefulness of a biosignature is determined not only by the *probability* that life produced it, but also by the *improbability* that nonbiological processes produced it. Biosignatures can be any observable phenomena such as elemental abundances, molecules, objects, isotopic abundance patterns, or processes that provide evidence of past or present life. Biosignatures include heteroatoms in graphitic carbon or isotopic patterns between reduced carbon and carbonates in ancient rocks (*e.g*., Bernard and Papineau, 2014), molecular biomarkers or their fragments (Summons *et al.*, 2008; Jolley and Douglas, 2012), fossil-like cellular structures (*e.g*., Schopf and Kudryavtsev, 2012), possible biogenic structures in diagenetic concretions, granules, and rosettes (Berner, 1968; Coleman, 1993; Papineau *et al.*, 2016, 2017), and microbially influenced structures such as stromatolite-like morphologies (*e.g*., Grotzinger and Knoll, 1999; Berelson *et al.*, 2011; Pepe-Ranney *et al.*, 2012) and Microbially Induced Sedimentary Structures (abbreviated as ‘MISS,’ Noffke *et al.*, 1996). It is important to note that biosignatures typically include some objective measure or indicator of normal biological processes (*e.g*., pathogenesis or photosynthesis) (Mata *et al.*, 2012), and these factors can be difficult to define, let alone measure (Cady *et al.*, 2003). One example of this challenge is identifying particular biological processes associated with potential global-scale biosignatures in exoplanets (Des Marais *et al.*, 2002).

An ‘abiosignature’ is a substance, object, or pattern that has a nonbiological origin. The usefulness of an abiosignature is determined not only by the *probability* that an abiotic process produced it, but also by the *improbability* that biological processes produced it. Definitive abiosignatures could provide insights about how an uninhabited habitable planet would appear when sampled directly or observed remotely. Characterizing abiosignatures should enhance our capacity to delineate and confirm biosignatures.

An ‘ambiguous biosignature’ (termed a ‘potential biosignature,’ Des Marais *et al.*, 2008) is a feature that occupies the ‘gray zone’ of uncertainty between biosignatures and abiosignatures. An ambiguous biosignature might compel investigators to gather more data before reaching a conclusion as to the presence or absence of life. Navigating this ‘gray zone’ is a central challenge for astrobiology life detection efforts.

‘Agnostic biosignatures’ are substances, objects, and/or patterns whose origins specifically require biological agents and also include features that might not have originated on Earth. Agnostic biosignatures compel us to envision attributes of life that are more fundamental and widespread in the cosmos than attributes that are apparent in our own biosphere (Johnson *et al.*, 2018; Exoplanet Science Strategy, 2018).

We address the challenges of differentiating between biosignatures and abiosignatures for astrobiology, and searching for the origins of life, by beginning within the perspective of the earthly bias that shapes present science. The interdependent linkages of biology, chemistry, and geology are fundamental to defining the following: (1) what constitutes a biosignature or a biomarker, or conversely an abiosignature; (2) how extant life would be recognized, preserved, and identified; and (3) whether fossil life-forms exist and can be detected and recognized elsewhere in the Universe.

Aside from identifying and measuring biosignatures across multiple disciplines, including biology, geology, engineering, and environmental science (Cady and Noffke, 2009), we need to define what constitutes a biosignature to search for evidence of life on other planets and moons, and attempt to constrain the timing of the origins of life on Earth. Extraterrestrial exploration for life is extremely challenging due to the technical demands of making remote measurements in the Solar System and beyond. Similarly, searches for the earliest traces of life on Earth are challenging due to ongoing disruption by the formation of tectonic/metamorphic belts, the constant recycling of the crust, Earth's active hydrologic cycle and consequent weathering and erosion, and the ubiquity of modern life.

## 3. Life Limits and Uncertainties

Beyond the challenges described above, there is a further complication in evaluating biosignatures. Science is presently unable to explain satisfactorily how terrestrial life originated, namely, whether early life and extraterrestrial life were or are compositionally or functionally similar with modern terrestrial life and had the same effects on the environment as modern life that we can observe directly. Biological evolution is undoubtedly influenced and constrained by larger scale planetary and Solar System processes, some of which are beyond biology's influence, such as tectonics (Lindsay and Brasier, 2002), solar activity (Ribas *et al.*, 2005), and impacts (Kring, 2000). Yet, in other large-scale processes, such as the terrestrial nitrogen cycle (Stüeken *et al.*, 2016; Laneuville *et al.*, 2018), biology may have become a major factor very early on. As an example of the intertwining of planetary and biological processes, it is widely assumed that the buildup of molecular oxygen in the Earth's atmosphere is due to biology (Des Marais *et al.*, 2002). However, the pacing of the rise of oxygen depended on parameters inherent in Earth's formation, for example, its size and elemental composition, which were inherited from stochastic processes during formation of the Solar System.

The structural complexity of organisms appears to have increased during the biological evolution on Earth, although it is acknowledged that there are multiple criteria by which complexity can be gauged (Emmeche, 1997; Hazen *et al.*, 2007). The earliest organisms were unicellular (Woese, 1998) and perhaps even preceded by acellular ones for which we have no fossil record. There was then a development from single-celled bacteria, archaea, and eucarya, to multicellular organisms. As a result of this progression, biochemical complexity has also evolved over time according to various metrics (Woese, 1998; Böttcher, 2018), with certain metabolic capabilities arising sequentially (*e.g*., oxygenic photosynthesis or oxidative metabolism). It further seems logical that, however, life began, it started in a “simpler” state that included less compositional, morphological, and functional capabilities (Woese, 1998). These differences could naturally affect the types of biosignatures a planet would be capable of producing at any given point in its history once life began.

**FIG. 1.**
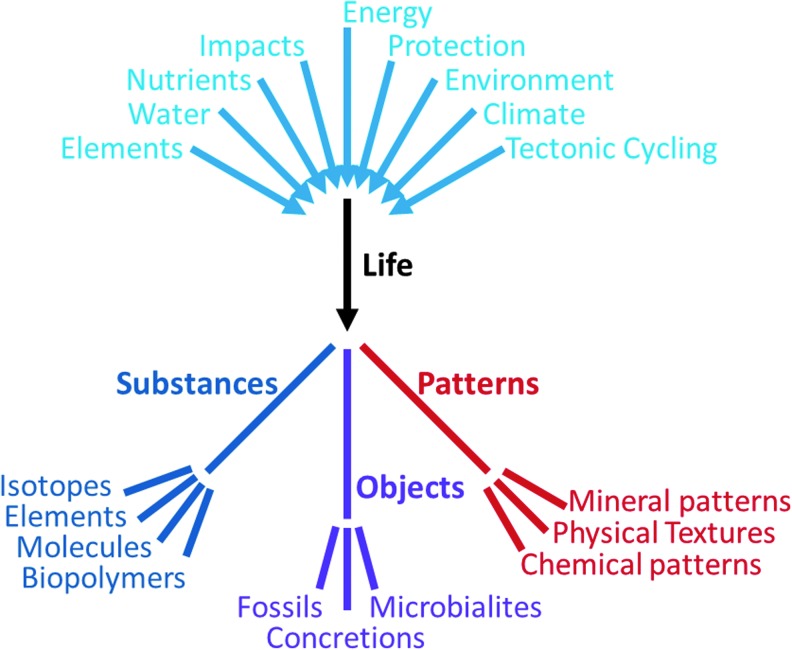
Tree diagram of the relationship of variables affecting the formation and development of life, and the resulting biosignatures in the three categories of substances, objects, and patterns. These major components and products capture the current state of opinion in astrobiology. Within each category, there are challenges to identify and measure the type of biosignatures, evaluate the fluxes that may be relevant to enhancing life, understand the context of scales and relationships, and evaluate importance, applicability, and confidence in the signature.

We presently lack a universal definition of life, which contributes to making the search for unambiguous biosignatures a central unifying challenge of astrobiology (see *e.g*., Smith, 2016). In this work, we assert that life uses environmentally available energy and matter (Fig. 1) to reproduce itself as both a structure and a process in a state of chemical and thermodynamic disequilibrium relative to the surrounding environment, and that life's processes generate waste and alter the environment. This alteration of the environment can result in simply changing it more rapidly than would occur in the absence of the catalytic properties of organisms or can produce phenomena that abiogenic substances, objects, and patterns may mimic.

The mutual interactions between life and its host planet may be self-reinforcing or self-amplifying. Indeed, it has been suggested that perhaps the entirety of the planet behaves as one large organism in some sense, with both biotic and abiotic spheres of influence overlapping across all planetary environments (Lovelock and Giffin, 1969). Direct evidence of such a complexity is not yet robustly in hand.

## 4. Cosmic Perspective

Our understanding of terrestrial life strongly clouds but uniquely informs the search for life beyond Earth. Terrestrial life has managed to obscure or overwhelm abiotic planetary processes to the point that Earth life is readily observable even from space (Sagan *et al.*, 1993). It is possible that a common stable outcome of the evolution of planetary biospheres is that a given biosphere may not be capable of entirely saturating its environment to the extent that life has on Earth. This idea was argued against by Lovelock and Margulis (1974) who favored all or nothing outcomes with regard to life dominating its host planet. Nevertheless, there may be transient periods during the development of biospheres in which the impact of biology on planetary processes is relatively feeble.

## 5. Biosignature Phenomena

**FIG. 2.**
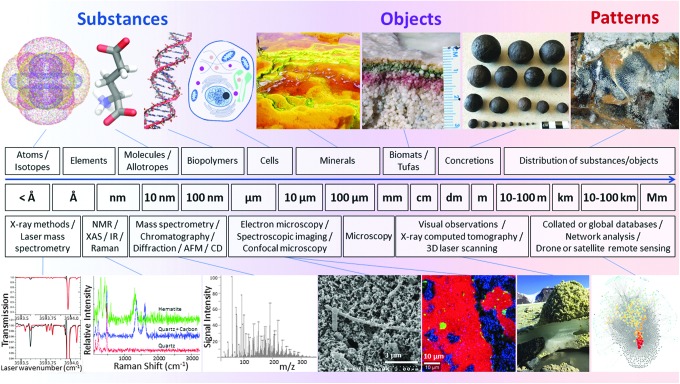
Biosignatures and life detection methods range from microscopic (left) to planetary scales (right). A nested astrobiological approach will provide context for the physicochemical parameters and processes governing the preservation of biosignatures. Images created by R.J.G. using VMD software (Humphrey *et al.*, 1996). Top row images: carbonate formation in Río Tinto (Fernández-Remolar *et al.*, 2012); biomats (Des Marais, 2003); concretions (M.A.C.); and biovermiculation patterns, Cueva de Villa Luz, Tabasco, Mexico (P.J.B.). Life detection analytical techniques in bottom row (left to right) are laser spectroscopy (modified with permission from Leshin *et al.*, 2013); Raman spectra from 3.49 Ga Dresser Formation chert (D.M.B.); high-resolution mass spectrometry (Parker *et al.*, 2016); scanning electron microscopy (Chivian *et al.*, 2008); Raman spectra map (D.M.B.), photograph of sulfur deposits on the Borup Fiord Pass glacier (Lau *et al.*, 2017) computational network analysis (http://dtdi.carnegiescience.edu) (Morrison *et al.*, 2017).

Based on our current knowledge, we discuss biosignature phenomena (including biomarkers) in three main categories: substances, objects, and patterns (Figs. 1 and 2). Each section includes a definition of the classification, the scales and methods of evaluation, and how the biosignatures can be validated or verified, with application to astrobiology. These three categories of biosignatures are complexly interrelated in that substances are present in objects and both can contribute to patterns. The challenges are to measure multiple types of biosignatures; to evaluate the fluxes that may be relevant to enhancing life (*e.g*., mass fluxes of elements, water, and nutrients as well as energy fluxes of protection, environment, and climate); and to understand the context of scales and relationships, with weightings of importance, applicability, and confidence in the signature.

### 5.1. Substances

Substances are materials, or combinations of materials, with structures that are fixed by chemical and physical constraints. Examples include elemental abundances, molecules, allotropes, enantiomers, minerals, and their associated properties. Geotemporal context can also help distinguish a biosignature from an abiotic substance. In this section, we explore criteria for unambiguous biosignatures and/or abiosignatures (or antibiosignatures of Walker *et al.*, 2018) stored in substances by the following: (1) examining substances or associations of substances that provide strong evidence for biological activity and therefore qualify as biosignatures; (2) addressing physical, chemical, and biological processes that preserve or degrade substances over time; (3) determining the spatial scaling and/or distributional relationships of substances required to map and validate biosignatures; (4) quantifying uncertainty in substance-based biosignatures to define a framework for their interpretation over time and space; and (5) exploring case examples.

#### 5.1.1. Substances as biosignatures

The search for life beyond Earth or in ancient rocks remains an exceedingly difficult problem (Tashiro *et al.*, 2017), in part, because of challenges in appropriately defining unambiguous biosignatures or abiosignatures (Westall *et al.*, 2015). Presently there is no fundamental framework or theory to evaluate the best substances or combinations of substances that might constitute a biosignature.

Many scientists have examined criteria required for life as we know it and then attempted to relate them to specific substances that might constitute direct or indirect evidence for life. Biogeochemical assemblages span scales from the least complex, most immutable units, such as elemental abundances, that can preserve evidence for metabolic processes, continuing up through greater levels of complexity. Alternatively, minerals and rocks constitute the base abiogenic matrix in planetary systems, yet minerals themselves and their morphology or diversity may be biosignatures themselves. Strong arguments can be made for substances as biosignatures (*e.g*., Catling *et al.*, 2018) especially when they occur in combinations, for example—minerals and isotopic patterns, minerals and morphology, organic molecules, and isotopic patterns. Hence multipronged approaches for biosignature detection are needed to address this complexity and interrelatedness.

#### 5.1.2. Preserving substances as biosignatures

**FIG. 3.**
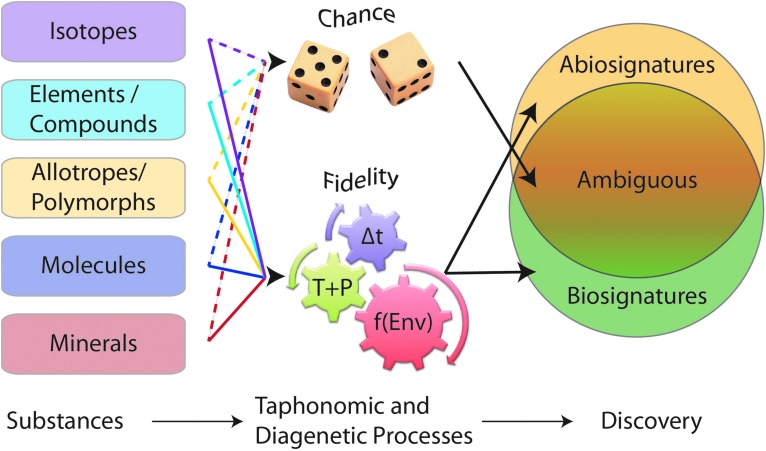
Most biosignatures in the geologic record are ambiguous, with discovery highly dependent on taphonomic and diagenetic processes. When these processes are not well understood, the resulting signatures are ambiguous, appearing to be the result of chance events (dashed lines). When processes are well understood, they function as high-fidelity representations of the original substances, biotic or not (solid lines). Multiple independent observations can distinguish the biosignature and abiosignature fields, reducing the size of the ambiguous signature field. Δt = time since formation of the substance. T+P = temperature and pressure changes the substance experiences over time. “f(Env)” = environmental conditions under which diagenetic and taphonomic processes occur, specifically, chemical reactions and the presence and movement of fluids or absence of fluids over time.

Changes in physicochemical conditions over time and space can produce significant ambiguity in identifying biosignatures (Fig. 3) (*e.g*., Farmer, 1999a).

To increase certainty, a purported biosignature is ideally interpreted in its original depositional and preservational context. In many cases, the original mineral or biological content of a depositional event is not preserved. Determining the extent and timing of preservational effects is not trivial and requires extensive comparison with natural systems and laboratory experiments (Grosch and McLoughlin, 2014, 2015). Unfortunately, there is often incomplete information about depositional and subsequent diagenetic conditions, and it is not always possible to accurately and experimentally model diagenetic processes. Hence, the likelihood of biosignature preservation, discovery, and accurate interpretation is limited, and a systematic approach to document the appropriate physicochemical conditions that best allow preservation is critical.

Radiolytic processing may be an important factor in biosignature preservation in planetary environments (Dartnell *et al.*, 2012; Pavlov *et al.*, 2012). Such a process can be part of the “f(Env)” term shown in Fig. 3, which broadly refers to environmental conditions under which diagenetic and taphonomic processes occur, especially chemical reactions and the presence or absence of fluids and their movements over time. Considering the multitude of potential mineral/organic combinations, how can the degradation products of radiation damage to organic molecules and minerals be recognized? Basic chemical principles can be applied to candidate combinations to identify potential reactions and develop tests for specific chemical, physical, and mineralogical products. The geologic and environmental context is important in all cases.

#### 5.1.3. Spatial scales and distributions to validate substances as biosignatures

**FIG. 4.**
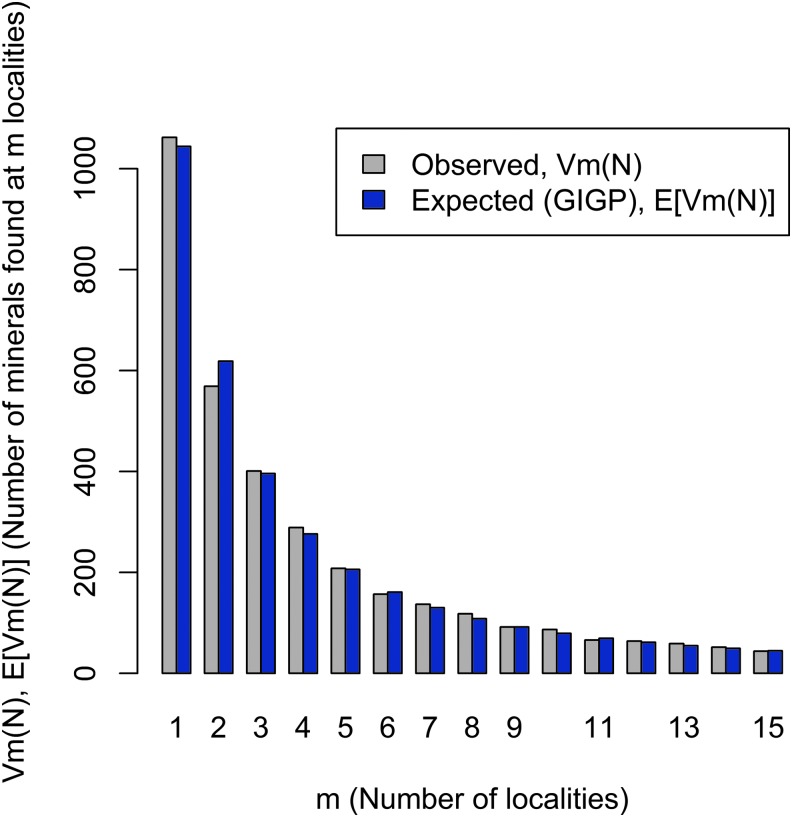
Earth's mineral inventory follows an LNRE distribution. Observed (gray) and modeled (blue) frequency distribution for rare minerals on Earth (Hazen *et al.*, 2015; Hystad *et al.*, 2015). Most of Earth's >5300 known mineral species are rare, occurring at ≤5 localities, and changes in Earth's environment caused by biology may contribute to this phenomenon. Statistical expected relationships GIGP means generalized inverse Gauss–Poisson distribution. LNRE, Large Number of Rare Events. Image: R.M.H.

The diversity and distribution of minerals at a planetary scale (*i.e*., a Large Number of Rare Events [LNRE] frequency spectrum) could itself be a biosignature. Recent analyses of large mineralogical data resources reveal that Earth's mineralogy conforms to a distinctive LNRE distribution (Fig. 4). This type of distribution arises when a few species are commonly found but most species are rare. In the case of Earth minerals, over half of all species are known from five or fewer localities (Hazen *et al.*, 2015; Hystad *et al.*, 2015).

**FIG. 5.**
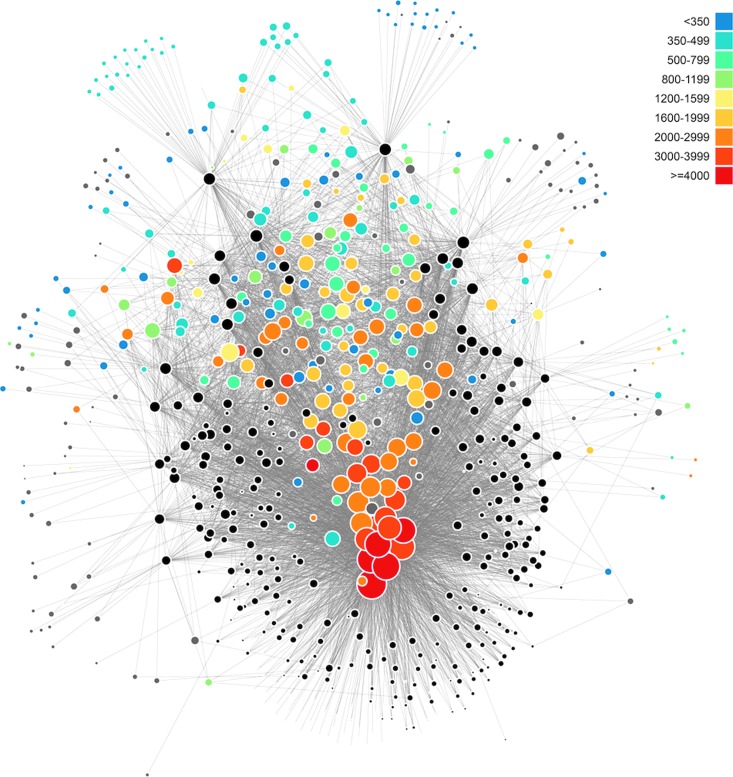
A bipartite network diagram for all carbon-bearing mineral species reveals relationships among mineral localities (represented by black circles), connected to mineral species (represented by colored nodes) that occur at those localities. Sizes of locality nodes indicate how many mineral species occur at that locality. Sizes and colors of mineral species nodes reflect mineral abundances. The topological distribution of mineral nodes represents an LNRE frequency spectrum (Fig. 4). Image: R.M.H.

The LNRE distribution is also manifest in network analyses of mineral systems (Morrison *et al.*, 2017). Consider the bipartite network for 400+ carbon-bearing minerals (Fig. 5). Large red nodes positioned near the center of the “U”-shaped array of black locality nodes indicate the most common species, whereas the “halo” of small blue nodes represents the large number of rare minerals found at only one or two localities. The topology of this network diagram is a visual representation of an LNRE mineral distribution.

Although data are available for >5300 minerals found in hundreds of thousands of locations on Earth (mindat.org), we lack comprehensive data for LNRE analyses of any other solar system body. Preliminary analysis suggests that the Mars and Moon have much lower mineral diversity than Earth, along with spatial distributions that do not conform to an LNRE model (Hazen *et al.*, 2015). Thus, while an LNRE distribution might constitute a global scale biosignature (Hazen *et al.*, 2015; Hystad *et al.*, 2015), for the foreseeable future, this hypothesis will be testable for only a few bodies in our Solar System.

#### 5.1.4. Energy production as a biosignature

The recent discovery of subsurface chemolithotrophic microorganisms living in a relatively low-radiation biosphere (*cf*. Colman *et al.*, 2017) suggests evolution of certain genes (*e.g*., hydrogenases, acetyl-CoA synthases, and CO-dehydrogenases) that are more prevalent in subsurface chemolithotrophic organisms than surface organisms (Colman *et al.*, 2017). Thus, the rock matrix supplies multivalent elements that can transfer electrons via reduction/oxidation (redox) reactions to produce energy in the system, and metabolism is accelerated with mobilization of these redox substrates by groundwater.

#### 5.1.5. Uncertainties in evaluating substances as biosignatures

Defining biosignatures must be weighed against the null hypothesis, which states that every known nonbiological process must be rejected before a biological conclusion can be adopted. For example, graphite in sedimentary rocks from hydrothermally influenced environments could have sourced nonbiological carbon formed from Fischer–Tropsch Type (FTT) synthesis during serpentinization. Consequently, we need to adopt uniform principles for evaluating biosignatures and consider analogs of biosignatures in younger rocks where biology has left a stronger trace (*e.g*., Dodd *et al.*, 2017). It may also be useful to develop methods to assign confidence to the variables described in Fig. 1. Such an approach occurs in ore deposit exploration, where expected formation processes are combined into a model with weights or ranks (*e.g*., Wyborn *et al.*, 1994; Skirrow *et al.*, 2009).

##### 5.1.5.1. Importance of abiotic chemistry

Searches for extraterrestrial life often focus on biomarkers but determining whether a specific compound is of extraterrestrial biological origin and not a terrestrial contaminant is the crux of this problem. Abiotic chemistry must be understood sufficiently in detail such that when a signal is observed, there is a reasonable certainty as to whether it is biotically or abiotically produced versus being a terrestrial contaminant (Fox and Strasdeit, 2017). Extensive work in abiotic chemistry shows that a variety of biochemicals can be generated by abiological processes, for example, through atmospheric (Miller, 1953), hydrothermal (Hennet *et al.*, 1992; Amend and Shock, 1998), or interstellar chemistry (Bernstein *et al.*, 2005). These studies caution that many seemingly complex or uniquely biogenic compounds may be at best ambiguous biosignatures and they could be called dubio-biosignatures (*e.g*., Cady *et al.*, 2003). Indeed, environmental chemistry that generates organic complexity is widely viewed as being a stage in the emergence of life (Cleaves, 2012), and thus, it is possible that such compounds could be markers of transitional stages in biogenesis, although not of life *per se*.

Increased knowledge of abiotic chemistry is also synergistic with biomarker detection. That is, knowledge of the conditions required for the emergence of life can inform the types of extraterrestrial environments where life should be sought. Conversely, extraterrestrial signals determined to be abiotic rather than biotic can also point to potentially novel naturally occurring abiotic chemistries and can provide insight into prebiotic chemical processes.

The transition from nonlife to life must have included a means to produce and complexify organic molecules. As an example of an organic-generating abiotic process, FTT synthesis involves the abiotic metal-catalyzed reduction of CO or CO_2_ by H_2_ to produce reduced carbon compounds. Depending on the availability of other compounds, these abiotic organic molecules can include methane, short-chain alkanes, carboxylic acids, and nitrogenous and sulfurous organic molecules, which may contribute to the synthesis of other prebiotic molecules (Rushdi and Simoneit, 2004; McCollom, 2016).

Once abiotically synthesized, other abiotic reactions may further process organic compounds. For example, the dicarboxylic acid, malonate, is oxidized by sulfate and bromate through a chemically oscillating, Belousov–Zhabotinsky (B-Z) reaction (Zaikin and Zhabotinsky, 1970). Such reactions are somewhat similar with metabolic reactions. Chemically oscillating reactions have been proposed as stimulants for the development of early metabolic pathways (Russell, 2003). In contrast with FTT reactions, chemically oscillating reactions are not known to produce organic compounds with ∂^13^C values similar with those of metabolism.

On modern Earth, there are few examples of unambiguously abiotic organic synthesis supported by carbon isotope data (*e.g*., Prokurowski *et al.*, 2008) (also see Section 5.3.2). The occurrence or prevalence of chemically oscillating or other types of abiotic reactions in nature and their potential for isotopic fractionation of carbon or other elements remain largely unknown, although they may be more common and widespread than currently recognized (Papineau *et al.*, 2016, 2017).

#### 5.1.6. Substance case examples

##### 5.1.6.1. Elements and compounds

Biology affects most major and minor elements on Earth's surface with respect to their abundance in various reservoirs and incorporation into molecular and mineral species. We explore the nitrogen and carbon cycles here, but effects are also evident in other biogeochemically active elements. For example, the enormous quantity of O_2_ in the modern atmosphere is almost entirely due to biological activity.

###### 5.1.6.1.1. The nitrogen cycle as a biosignature

Nitrogen is abundant in Earth's atmosphere as N_2_, which is difficult to fix abiotically (due to the strength of the N-N triple bond). The evolution of metabolic pathways to fix atmospheric nitrogen for use in biomolecules such as DNA and proteins has allowed life to thrive despite the relatively low flux of abiotically fixed nitrogen (Falkowski, 1997). As the oceans and atmosphere became suffused in O_2_, life developed a variety of pathways to cycle nitrogen back to the atmosphere, the most efficient being biological denitrification, which displays marked isotopic fractionation (Nielsen, 1992; Sigman *et al.*, 2009). As N is recycled in ecosystems, life greatly augments the amount of N_2_ drawn down from the atmosphere and alters the way that fixed higher and lower oxidation state N-species can be passed into the mantle by subduction (Zerkle and Mikhail, 2017; Laneuville *et al.*, 2018).

In Precambrian sedimentary rocks, N isotopes in graphite, kerogen, ammonium-bearing phyllosilicate minerals, and bulk rock are interpreted variably. They may be seen as possible signatures of either biological nitrogen fixation or ammonium assimilation when ^15^N-depletions occur or attributed to denitrification when ^15^N-enrichments occur (Thomazo and Papineau, 2013). However, nonbiological processes such as diagenesis, metamorphism, fluid/rock interactions, and possibly varying atmospheric N-isotope composition can add significant uncertainty to the interpretation of the fractionation origin (Ader *et al.*, 2016).

###### 5.1.6.1.2. Carbon cycle

**FIG. 6.**
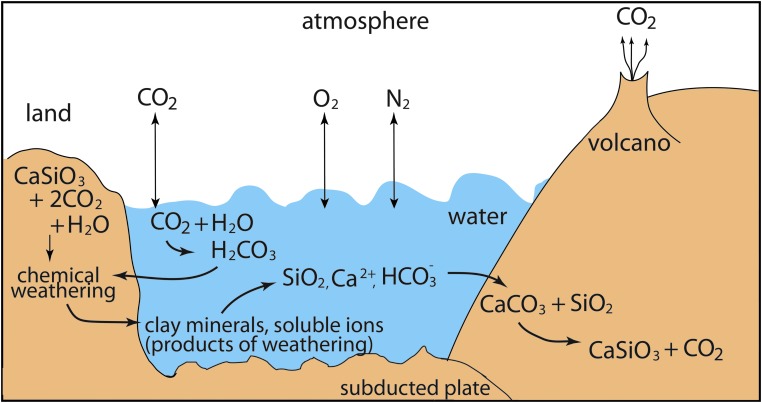
Complex linkages of the C and Si cycles (Kasting and Catling, 2003). Atmospheric CO_2_ dissolves in surface waters. The dissolved and atmospheric CO_2_ is in equilibrium. Dissolved CO_2_ reacts with water to form H_2_CO_3_ (carbonic acid, a weak acid). H_2_CO_3_ dissociates into H^+^ and HCO_3_^−^. Ultimately, H^+^ and water react with most common minerals, silicates, and carbonates, altering those minerals. The predominant weathering products are clay minerals (silicates) and soluble ions (Ca^2+^, Fe^2+^, Na^+^, K^+^). HCO_3_^-^ also remains in solution. Image: M.S.-R.

Transfers between air and other reservoirs, such as the biosphere, the oceans, and Earth's interior, control the concentration of CO_2_ in the atmosphere (Fig. 6). During oxygenic photosynthesis, plants, photosynthetic algae, and bacteria use energy from sunlight to combine CO_2_ with H_2_O to form carbohydrates (CH_2_O). These carbohydrates are used as an energy source, and O_2_ is released as a by-product. Some of the carbohydrate is stored as biomass. Consumers such as animals, fungi, and bacteria get their energy from this excess biomass via respiration, in which O_2_ is combined with carbohydrates to liberate energy, with water and CO_2_ as by-products.

###### 5.1.6.1.3. Carbonate versus silicate formation on Earth

Carbon and silicon cycles are linked by chemical weathering and biological stoichiometry (Wang *et al.*, 2016) (Fig. 6). Some geochemical factors (*e.g*., thermodynamics, chemical kinetics, hydrology, host-rock mineralogy, and texture) affect silicate and carbonate mineral formation and degradation. The microbial carbon/silicon “cycle” on early Earth would likely have involved ultramafic rock and therefore increased magnesium values rather than the low magnesium carbonate forming today (Power *et al.*, 2013).

Several studies consider the chemical products of autotrophic and heterotrophic microbial metabolic processes (Castanier *et al.*, 2000; Bennett *et al.*, 2001; Sánchez-Román *et al.*, 2008; Power *et al.*, 2011; Pace *et al.*, 2016) in addition to the chemical requirements for the formation of carbonates and other authigenic minerals (*e.g*., Sánchez-Román *et al.*, 2014; Ruff and Farmer, 2016). Most of these studies, which are based on field observations and validated by laboratory experiments, explore the role of microbes in mineral formation. Indeed, results suggest that the range of inorganic changes in the conditions alone is insufficient to induce mineral precipitation. Consequently, these studies suggest that biological processes must play a major role in mineral precipitation. Carbonate minerals could possibly comprise a biosignature when taken in the geologic and atmospheric context.

##### 5.1.6.2. Organic molecules

###### 5.1.6.2.1. Nucleic acids

Almost all known terrestrial life uses DNA as its genetic material, except for some RNA viruses. Of course, it is arguable whether viruses are living in the same sense that other organisms are alive (Lai and Cavanagh, 1997). It is possible that extraterrestrial biology could use an alternative information storage molecule (Pinheiro *et al.*, 2012; Cleaves *et al.*, 2015), and whether evolutionary processes would universally result in the same biochemistry is unknown. It may be reasonable to begin searching for living systems that we are familiar with, and detection of unambiguously extraterrestrial nucleic acids could provide a strong biosignature.

Detection of nucleic acid biomarkers in extraterrestrial environments, for example, Mars, could indicate whether a signal came from organisms with common ancestries to those on Earth (Mojarro *et al.*, 2017; Pontefract *et al.*, 2017). The abundance of nucleic acid building blocks produced in the cosmos may have pushed all life to use nucleic acids as genetic materials (Callahan *et al.*, 2011); thus, using nucleic acids as extraterrestrial biomarkers may provide an unambiguous biosignature. Still, there are multiple known ways that the environment can make the compounds that comprise nucleic acids.

###### 5.1.6.2.2. Amino acids and peptides

Peptides are another major class of biopolymer present in all extant life on Earth. These are polymers of a limited set of 20 common amino acids, and importantly, in terrestrial life, amino acids are exclusively l-enantiomers (except glycine, which is achiral). How life evolved to use specifically this set of amino acids or enantiomers is unknown (Blackmond, 2010; Ilardo *et al.*, 2015). Although extant life only uses 20 proteinogenic amino acids, many of which have been found in extraterrestrial samples (Kvenvolden *et al.*, 1970), extraterrestrial examples of proteinogenic amino acids occur alongside many other types of nonproteinogenic amino acids (Cronin and Pizzarello, 1997; Ambrogelly *et al.*, 2007). Nonproteinogenic amino acids are, by definition, not found in proteins although some are found in natural products (Walsh *et al.*, 2013). The observation of proteinogenic amino acids by itself does not constitute a biosignature, as abiotic processes can also form these molecules (Miller, 1953; Mullen and Sutherland, 2007; Aubrey *et al.*, 2009; Higgs and Pudritz, 2009), and even peptides are not necessarily biosignatures, as they can also be formed abiotically (Leman *et al.*, 2004; Danger *et al.*, 2012; Kitadai *et al.*, 2017). However, the exclusive detection of extraterrestrial homochiral peptides of significant length is not probabilistically favorable and may be an unambiguous biosignature (Orgel, 1998). This raises questions of whether homochiral peptides might be produced abiotically from a racemic pool of abiotically produced amino acids (Mathew *et al.*, 2004; Córdova *et al.*, 2005; Meierhenrich *et al.*, 2005).

Still, recent studies have shown that functional (Mohamed *et al.*, 2017) d-peptides can be produced biotically (Katoh *et al.*, 2017). Perhaps large quantities of either homochiral peptides, or even the coexistence of both types of homochiral peptides (but not mixed chirality peptides), observed beyond the Earth would be indicative of extraterrestrial life. Unfortunately, biopolymers tend to degrade over time, and the structural information that may allow them to serve as biosignatures can be lost over relatively short time periods.

###### 5.1.6.2.3. Distribution of molecules

When the isotopic or structural information of a molecule is not sufficiently diagnostic of its origin, the relative concentration of the molecule in the environment compared with other chemically related molecules may instead be used as a biosignature. Due to thermodynamic and kinetic constraints on the rate of formation of molecules during abiotic synthesis, a continuous spectrum of molecules, enriched in kinetically allowable low-molecular-weight compounds, is expected. For example, hydrocarbons synthesized via FTT processes are characterized by an exponential decrease in abundance with increasing number of carbon atoms (Sherwood Lollar *et al.*, 2002). This is in stark contrast to biological systems where metabolism results in the synthesis of only a specific subset of compounds.

Through enzymatic catalysis, organisms can rapidly synthesize compounds, even those that require a high energy of formation, because of the evolutionary benefits imparted by their synthesis (Dorn *et al.*, 2011). The principle that biological metabolism uses a discontinuous subset of biochemicals is hypothesized to be universal to all forms of life (McKay, 2004; Davies *et al.*, 2009). On Earth, uneven distribution patterns suggestive of biological origins are particularly evident in larger organic molecules where biosynthesis uses two-carbon building blocks, for example, in the case of enriching fatty acids of even carbon number in the environment (Botta *et al.*, 2008).

###### 5.1.6.2.4. Allotropes

Some elements and minerals can exist in more than one kinetically stable form in the same physical state (solid, liquid, or gas). For elements, these forms are known as allotropes. Examples of allotropes include diamond, fullerene, and graphite for carbon, molecular oxygen (O_2_) and ozone (O_3_) for oxygen, and the numerous types known for sulfur, including various cyclo and catena allotropes. Since one allotrope may be more kinetically favored by a biological synthesis mechanism over an abiotic mechanism, the relative abundances of these may serve as a biomarker.

###### 5.1.6.2.5. Cells/compartments

Common chemical constituents characterize terrestrial life. The interactions of these constituents define living systems and, for biochemical reactions to occur over appropriate timescales, the concentration of biomolecules must be relatively high (Matsuura *et al.*, 2012; Sunami *et al.*, 2016). This is generally achieved through the formation of cellular and subcellular compartments. Life uses a range of compartmentalization techniques, from the membraneless stress and p granules and nucleoli (Montgomery, 1898; Brangwynne *et al.*, 2015) to a range of membrane-bounded organelles and cells themselves. The organization of cells is not uniform. For bacteria and archaea, nuclei are absent. Most eurkaryotes have a single nucleus, some, for example, *Bryopsis plumosa* (Kim *et al.*, 2001) and *Caulerpa prolifera* (Kaplan and Hagemann, 1991) are multinucleate giant cells.

The different possibilities for the emergence of membrane-based compartmentalization have led to a significant research effort to build prebiotically plausible synthetic cell analogues that are capable of mimicking certain aspects of extant life (Szostak *et al.*, 2001; Kurihara *et al.*, 2011; Kuruma, 2015; Trantidou *et al.*, 2017). Analogs demonstrating metabolism, growth, replication, division, and evolution have been devised in the laboratory. These research efforts not only describe plausible options for the earliest forms of life on Earth but also lead to questions of how life can be defined in general terms and pose questions about the kind of compartments and their components that could be considered unambiguous extraterrestrial biosignatures.

##### 5.1.6.3. Mineral biosignatures (and abiosignatures)

Relatively little focus has been applied to using minerals as biomarkers. Mineral speciation and mineral morphology are two phenomena that might be used as biosignatures to reveal an extant or fossil biosphere.

###### 5.1.6.3.1. Mineral species as biosignatures

Earth boasts >5300 approved named mineral species, each with a unique chemical composition and crystal structure (rruff.info/ima). About 1500 of these diverse minerals can be unambiguously shown to originate through nonbiological, igneous, or metamorphic processes. In addition, hundreds of alteration minerals formed by hydration reactions, or species formed through evaporation of saline solutions, may occur on nonliving worlds (Hazen *et al.*, 2008; Hazen and Ferry, 2010; Hazen, 2013). As noted earlier, all purported biosignatures must be evaluated in the environmental context, and mineral species are no exception. Consequently, the occurrence of these species alone cannot be used to claim a biological origin.

In contrast, two-thirds of known mineral species on Earth arise directly or indirectly through biological alteration of the near-surface environment. Most abundant among these are minerals formed through the oxidative alteration of other minerals, notably thousands of oxidized minerals contain multivalent elements sensitive to oxidation/reduction, including transition metals (*e.g*., Cu, Fe, Mn, Ni) and metalloids (*e.g*., As, Sb), and nonmetals (*e.g*., C, S). Some minerals, such as the microbial precipitate hazenite [KNaMg_2_(PO_4_)_2_·14H_2_O] (Yang *et al.*, 2011), arise exclusively through biological activity. In addition, over 60 organic minerals, including oxalates, hydrocarbons, derivatives of guano, urinary tract minerals (*e.g*., struvite, NH_4_MgPO_4_·6H_2_O) (Sánchez-Román *et al.*, 2007), and one geoporphyrin (abelsonite; NiC_31_H_32_N_4_), are unambiguously the by-products of biological activity (Hazen *et al.*, 2013).

Can mineral species that appear to be unambiguously biological on Earth occur through purely physical and/or chemical processes on other planets and moons? There are a few thousand minerals that arise from oxidative weathering—presumably the consequence of oxygenic photosynthesis on Earth, but that might form abiotically on more oxidized worlds. Similarly, hydrocarbon minerals on Earth are usually associated with coal and other carbon-rich deposits assumed to arise from geologically modified or decayed biomass. Hydrocarbon minerals likely arise from purely physical and chemical processes on Titan (Cornet *et al.*, 2015). Except for a few distinctive organic minerals derived from complex biomolecules (Table 1), it is not yet possible to point to a single mineral or suite of mineral species that provide unambiguous evidence for an extant or fossil biosphere on another planet or moon. Thus, by themselves and without other biosignatures from organic molecules, isotopic and elemental compositions, such as mineral assemblages, are only “permissive” evidence.

**Table 1.** Organic Mineral Species Unambiguously Derived from Biomolecules

*Mineral*	*Formula*	*Biological source*
Abelsonite	NiC_31_H_32_N_4_	Chlorophyll-derived porphyrin
Guanine	C_5_H_3_(NH_2_)N_4_O	DNA/RNA
Oxammite	(NH_4_)_2_(C_2_O_4_)H_2_O	Derived from guano
Tinnunculite	C_5_H_4_N_4_O_3_·2H_2_O	Uric acid dihydrate; guano
Urea	CO(NH_2_)_2_	The principal component of urine
Uricite	C_5_H_4_N_4_O_3_	Uric acid; metabolic breakdown of purines

###### 5.1.6.3.2. Mineral morphologies as biosignatures

The most familiar and convincing mineral biosignatures are morphological in character. Biomineralized shells, teeth, and bones composed of carbonate, silica, or phosphate minerals retain obvious evidence of biological function. Stromatolites, burrows, and other trace fossils, coprolites, and other macroscopic fossils also provide convincing morphological biosignatures preserved in mineralized structures. Microscopic fossils such as diatom frustules, amebic tests, radiolarian skeletons, plant biominerals (phytoliths), and more also are distinctively biological. Many of these mineral morphologies are also treated as objects below.

In a few cases, the morphology of individual crystal grains may point to an unambiguous biological origin, such as microbially precipitated minerals that display morphologies not otherwise likely to occur. Uraninite (UO_2_) is an interesting mineral example whose morphology appears to have changed through deep time as a consequence of biology (Hazen *et al.*, 2009). In Archean rocks before the Great Oxidation Event (GOE), uraninite is typically coarse grained, occurring abiotically in both igneous formations and as stream-eroded grains in sediments (Rasmussen and Buick, 1999). However, more recent formations display concentrations of nanouraninite by strains of *Geobacter*, *Desulfovibrio*, and *Shewanella*, which may couple acetate oxidation to the reduction of aqueous uranyl cations, UO_2_^2+^ to nanouraninite (Lovely *et al.*, 1991; Spear *et al.*, 2000; Fayek *et al.*, 2005; Long, 2008; Sharp *et al.*, 2008). Although nanouraninite is still ambiguous as a biosignature, the role of microbes in modifying mineral morphology represents an important opportunity in future biomarker research.

##### 5.1.6.4. Chemical disequilibrium

**FIG. 7.**
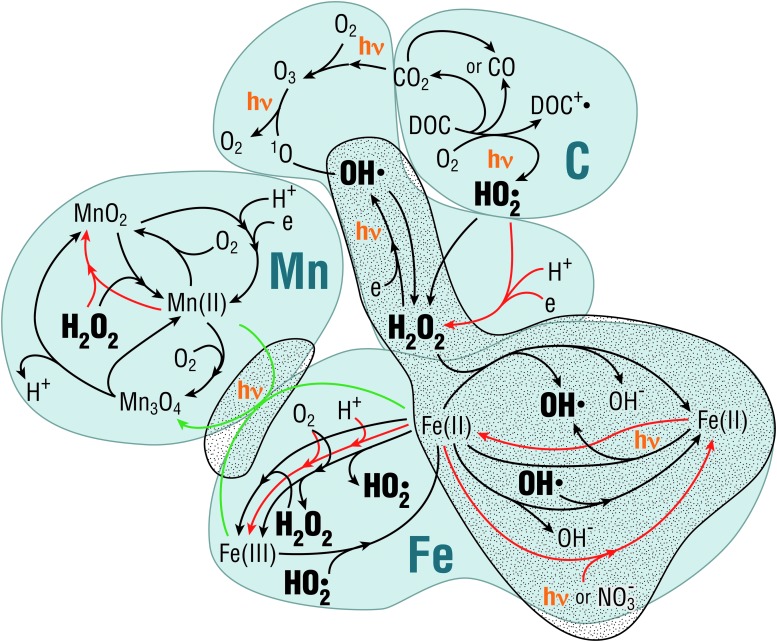
Linked biotic and abiotic processes illustrate formation and degradation of reactive oxygen species in aquatic systems, including photochemical processes. Three systems are used to illustrate the processes: carbon, which is the dominant mechanism in most aquatic systems, manganese, and iron (*cf.* Wilson *et al.*, 2000; Duesterberg *et al.*, 2008; Doane, 2017). Black arrows are abiotic reactions. Red arrows are biotic reactions. Green arrows are inferred reactions. The stippled area shows reactions that occur in the absence of oxygen. Dots by chemical compounds indicate an unpaired electron, that is, free radicals. Image N.W.H.

The cycle of reactive oxygen species (ROS) provides an example of linked abiotic and biotic processes that may help to distinguish biotic from abiotic conditions (Fig. 7). ROS are produced in the environment in the forms of hydrogen peroxide (H_2_O_2_), hydroxyl radical, and superoxide, among others. The abiotic ROS cycle produces chemical disequilibria in redox states of some transition elements, notably Fe and Mn (*e.g*., Doane, 2017).

In most modern surface waters, the main abiotic pathway for ROS production involves photoreactive dissolved organic matter. Yet, reactions involving multivalent elements are likely the most relevant for early Earth and planetary systems (Ramesh *et al.*, 2016; Doane, 2017 and references therein). ROS production could have been much higher on early Earth before oxygenation of the atmosphere because of the absence of an ozone layer (and thus a high flux of intermediate wavelength ultraviolet radiation [UVB: 280–315 nm]) and the higher concentrations of reduced multivalent elements in near-surface environments. These reactions cycle elements abiotically through redox states, leading to accumulation of ROS depending on the rates of formation and degradation.

The formation and degradation rates of ROS depend on intrinsic rate constants, temperature, pressure, and the concentrations of reactants. All ROS are damaging to life, which has developed mechanisms to detoxify H_2_O_2_ and superoxides although not able to detoxify hydroxyl radicals. Microbes rapidly degrade H_2_O_2_ and superoxide (Wilson *et al.*, 2000), limiting the maximum concentration achievable in surface waters. In the absence of microbes, it is possible that H_2_O_2_, and superoxide concentrations (in the presence of O_2_) would continue to increase until the rate of the degradation equals the rate of formation, which could lead to very high concentrations of environmental H_2_O_2_ and superoxide. Thus, high photochemically achievable concentrations of ROS could mean that there is no life, while lower but constant maxima could mean that life is present. Such a subtle distinction would require a highly nuanced interpretation. For example, Meadows *et al.* (2018) compare abiotic mechanisms of O_2_ production on exoplanets with the early emergence of photosynthesis as a source of O_2_ on Earth. The environmental conditions comprising planetary atmospheres and stellar spectra, under several models of such conditions, could produce biosignature false positives.

Krissansen-Totton *et al.* (2018) argue that the presence of disequilibria, specifically in carbon-bearing gases, CO_2_ and CH_4_, is a potential biosignature that can be detected in planetary atmospheres. Indeed, these two species of carbon would not be expected to occur together, and both can be detected remotely. They point out, though, that photochemical processes, among others, must be considered, particularly with respect to photodissociation of methane. The abiotic, and potentially biotic, rates of the formation and degradation reactions are key for allowing the concentrations of either carbon dioxide or methane to build up. Photochemical processes, particularly in the presence of suitable catalysts (*e.g*., Habisreutinger *et al.*, 2013), have the potential to put environmental pressure on planetary systems and maintain disequilibria.

### 5.2. Objects

The term ‘objects’ describes physical features produced by life, such as fossils, microbialites (in particular stromatolites), and biotextures as well as some physical features that are inferred to be related to life products (visible or invisible), such as concretions. Life is embedded in its environmental context and spatial scales are important for interpreting biogenicity. Characteristics from the macroscale down to the submicron scale need to be accounted for as much as possible although typically not all present in a given specimen, feature, or material type. Identifiable morphologies of potentially habitable environments at larger scales are visible with current instrumentation on Mars (*e.g*., orbiters); however, most are still ambiguous without the ability to evaluate features at smaller scales. Multiple methods examining features at multiple scales are needed to adequately characterize samples or systems to increase the level of certainty that a record of life is preserved. Spatial distributions of potentially biogenic features must also be characterized to understand context and move toward more certainty when identifying biosignatures.

Objects can be produced by organisms over a variety of scales, from macroscopic stromatolites (*e.g*., Awramik and Buchheim, 2015; Suosaari *et al.*, 2016) down to submicron biominerals, such as microbially generated magnetite (*e.g*., Stolz, 1993; Stal, 2012). Extreme environments are often thought of as the most likely sites for the origins of life on both Earth and Mars. However, it is important to remember that organisms adapt to their environment during evolution, and the origins of life may occur under milder conditions (Cleaves and Chalmers, 2004).

#### 5.2.1. Objects as biosignatures

Object biomarkers can have varying degrees of ambiguity with respect to their biogenicity. For example, bones are unambiguous biosignatures at the macroscale, because they can only be formed by vertebrates. However, macroscale textures in sedimentary rocks are potentially more ambiguous, and can form either biotically, as in the case of MISS (Noffke *et al.*, 2008) and stromatolitic lamination (Lee *et al.*, 2000), or by abiological physical processes, such as fluid flow or turbulence (McLoughlin *et al.*, 2008; Bower, 2011; Menon *et al.*, 2016), and abiological chemical processes, such as the abiotic precipitation of calcium carbonate (Pope and Grotzinger, 2000; McLoughlin *et al.*, 2008). To further complicate matters, the formation of stromatolitic lamination can involve a combination of abiotic and biotic processes (*e.g*., Riding, 2008; Suosaari *et al.*, 2016; Tosti and Riding, 2017). Continued investigation of the abiotic and biotic factors that can lead to stromatolite formation is necessary to discriminate their variable contributions (Awramik and Grey, 2005).

At smaller scales, morphological microbial body fossils can provide strong fossil evidence (Levett *et al.*, 2016) or be ambiguous, since many of the same mineral species involved with fossilization also occur in abiotic systems, and even mineral morphologies can mimic microbial ones in ancient rocks (*e.g*., Bower *et al.*, 2015, 2016; Crosby and Bailey, 2018). In addition, many of these body fossils have different morphological identification features at a variety of scales, which when observed collectively reduce the uncertainty of interpretation. The consensus is that multiple lines of evidence that combine chemistry, morphology, geologic context, and other features at different scales are required to determine biogenicity with confidence. Herein, we describe objects that can potentially be used to infer a record of biological activity on a variety of scales (from the large scale down to the small scale), followed by a brief discussion of biosignature preservation.

##### 5.2.1.1. Kilometer-scale context

Individual objects as biosignatures do not occur at large regional scales, except for some stromatolites. Massive stromatolite beds, a few to several meters thick, can be traced over ∼1000 km in the Mesoproterozoic Atar Group, Mauritania (Bertrand-Sarfati and Moussine-Pouchkine, 1988).

One of the first requirements in the search for past records of life is recognizing environments capable of high bioproductivity, long-duration habitability, and high preservation potential (Hays *et al.*, 2017). This is a critical first step in the search for a recognizable biosignature, although the topic of habitable environments is too large to be covered in detail here.

To recognize km-scale environments conducive to habitability and preservation of biosignatures, we need to be able to interpret the past or present habitability of environments. For example, indicators of such environments include clays (*i.e*., hydrated aluminosilicates with layered crystal structures) and carbonate lithologies combined with diagnostic large-scale morphologies such as layered rocks or deltas from orbital data in the context of Mars. Especially, attractive environments include exhumed and exposed sedimentary units, exposed subsurface deposits, spring environments, and regions where mafic rocks and sediments are layered over sulfate-rich sedimentary rocks (as sulfates record both the presence of water and contain sulfur—a potentially good energy source).

Subsurface environments are good candidates for having hosted continuous and long-lived potentially habitable environments—particularly the subsurface of Mars, and the liquid interiors of Europa and Enceladus presumably overlying rocky centers. The subsurface of a rocky planet such as the Earth often contains redox gradients at a variety of spatial scales, which could provide energy for chemolithotrophs on Mars (Boston *et al.*, 1992). In addition, these redox gradients and the potential for chemolithoautotrophy would likely involve reactions that create rapid mineral precipitation (*e.g*., the creation of iron-bearing, sulfate, or carbonate diagenetic cements) that increases the probability for preservation of life.

Examples of exposed subsurface environments on Mars include Margaritifer Terra where chaotic terrain is hypothesized to have resulted from expulsion of subsurface fluid (*e.g*., Carr, 1979; Thomas *et al.*, 2017). In addition, raised ridges that are resistant to erosion relative to the surrounding rock have been interpreted as possible examples of subsurface mineralization that has preferentially cemented these fractures, rendering them harder than the rest of the unit (Thomas *et al.*, 2017).

The environments of springs on ancient Earth and Mars have similarly high probabilities for both production and preservation (Hays *et al.*, 2017). Springs may not have the longevity of some other environments but may present ephemeral refugia (on geologic timescales) for life to survive inhospitable conditions, such as the Late Heavy Bombardment on Earth and increasingly inhospitable surface conditions during the Hesperian period on Mars. Rapid mineral precipitation can entomb microbes in these environments and preserve biogenic features over geologic timescales (*e.g*., Potter-McIntyre *et al.*, 2017).

A specific example of mafic deposits over layered sulfates (such as N.E. Syrtis on Mars) (Ehlmann and Mustard, 2012) may represent habitats that have excellent production and preservation potential. On Earth, analog research on mafic intrusions into sulfate-rich sedimentary rocks shows that these environments are promising astrobiological targets (Foster *et al.*, 2010). These are subsurface environments that would be locally sterilized during mafic emplacement. However, the fluid accompanying the mafics would mobilize sulfur and other bioavailable elements to supply the environment with fresh reactants for metabolism. Some degree of sterilization could create an ecological niche for organisms and an environment rich with nutrients, increasing the chances for high biological production and high preservation due to rapid mineral precipitation.

The potential for kilometer-scale contexts described above can support more insightful interpretations of meter-scale and smaller objects than analyzing those objects alone. In the absence of such bridging information, isolated objects may be more ambiguous and subject to multiple interpretations. The more subtle the smaller object or feature, the more the kilometer-scale view of the environment can contribute critical information.

##### 5.2.1.2. Meter-scale objects

Meter-scale biosignatures can include microbialites (stromatolites), microbially induced sedimentary structures (aka MISS) on extensive bedding plane surfaces, and concretions that are visible to the naked eye and are often spatially distributed from the meter to the hundreds of meters scale (*e.g*., Awramik, 1992; Noffke *et al.*, 2008; Potter *et al.*, 2011; Chan *et al.*, 2012; Fralick and Riding, 2015; Potter-McIntyre *et al.*, 2017). Some macroscale features represent the collective physical remains of structured microbial communities (Awramik, 1992), where diagnostic wavy laminar, domical, clotted, conical, branching, and stratiform morphologies would not form in such finely laminated deposit were it not for the microbial influences. Indeed, in Archean rocks, the macrostructures associated with microstructures (*e.g*., stromatolitic lamination and MISS) can be extensive and compelling as a morphological biosignature (Noffke *et al.*, 2008; Noffke and Awramik, 2013). Stromatolites and certain types of microbial mats are further discussed in a later section on patterns.

Trace fossils (ichnofossils) record the activities of a variety of different types of organisms and are visible at the submeter scale. Common terrestrial trace fossils include footprints, burrows, root traces, and imprint textures. For example, charophytes (freshwater algae) can leave characteristic imprints in rock that consist of 1 mm by 5–10 mm shallow (<0.5 mm) vugs (Potter-McIntyre *et al.*, 2014). Some evidence suggests that syneresis cracks are biological in origin and can also be considered microbial trace fossils (Harazim *et al.*, 2013; Mariotti *et al.*, 2014). Other microbial trace fossils also occur at micron scales in the form of mineral precipitation patterns or microborings (Staudigel *et al.*, 2015; Nikitczuk *et al.*, 2016).

Taken collectively, these textures can provide clues about paleoenvironments and the types of communities that inhabited them. However, physicochemical changes to sedimentary deposits over geologic time can also result in the formation of similar abiotic textures in ancient rocks (*e.g*., Grosch and McLoughlin, 2015; Davies *et al.*, 2016). For example, mudcracks can resemble burrows in a cross-sectional view, or clotted paleosol development can resemble burrow textures. Larger organisms and collections of organisms (biofilms, microbial mats) may be preserved but may be significantly reduced in size due to compaction during diagenesis (Bower *et al.*, 2017) and dewatering. Consistency in parameters such as texture, size, and orientation can be useful in differentiating biotic from abiotic signals but examining these features at a variety of scales is still necessary to deduce biogenicity in ancient rocks.

##### 5.2.1.3. Centimeter-scale objects

**FIG. 8.**
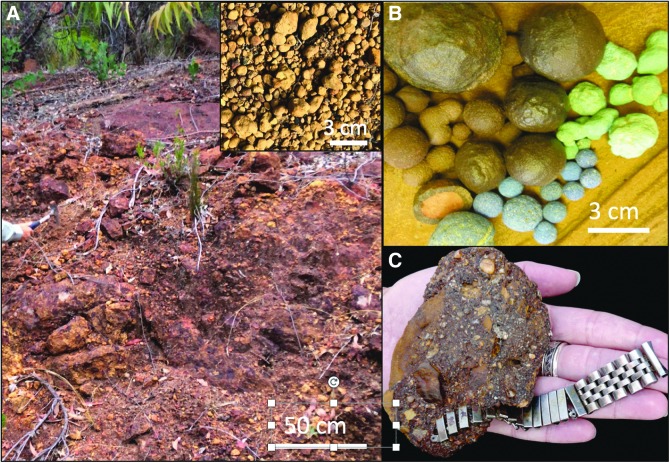
Iron oxide mineral precipitates have various biomediated to ambiguous origins. **(A)** Bauxitic paleosols of the Yilgarn Craton, Western Australia, show deep weathered zones, heavily influenced by plants to microbes and bacteria. Upper right inset shows loose paleosol pisolite (pisoliths) with various microbial forms (Anand and Verrall, 2011). **(B)** Concretions of goethite (brown), malachite (green), and azurite (blue) mineralogies from Utah are more ambiguous in their origins, and lack any fossil nuclei. **(C)** Iron oxide concretions around human-made objects (*e.g*., metal watch band from the Chesapeake Bay) suggest rapid, biomediated cementation on the orders of years. Images **(A, B)** M.A.C.; image **(C)** S. Godfrey, supplied by R.M.H.

Obvious, identifiable centimeter-scale fossils need little explanation of their biogenicity because we have a great deal of contextual knowledge about life on Earth, but large putative fossil materials on other planets may be much more difficult to interpret or even notice. Carrying this idea further, a range of other cm-scale objects could contain biosignatures similar with microbialites but perhaps even on finer microscopic scales. This range could include nonskeletal carbonate grains such as coated grains, including oncoids, ooids, pisolites, and others (Flügel, 2010). Iron oxide nodules of Earth's near-surface critical zone can exhibit a suite of varieties from those with clear biosignatures (*e.g*., hematite and goethite paleosol mineralization that preserve fruiting bodies, fungi, organic matter, and bacteria; Anand and Verrall, 2011) (Fig. 8A), to others that are ambiguous at best (Fig. 8B, C).

Concretions (Fig. 8B) are diagenetic, cemented mineral masses that comprise another example of centimeter-scale objects (although concretions can vary from meter- to millimeter-scales) with or without clear relationships to identifiable fossils (*e.g*., Raiswell *et al.*, 2000; Mozley and Davis, 2005; Potter *et al.*, 2011). Concretions without any obvious fossil nuclei are often assumed to be products of physical cementation, although it may just be a problem of recognizing subtle biosignatures.

If all of Earth's surface to subsurface waters has harbored microbes throughout most of the rock record, then biomediation must be considered possible for any authigenic mineral, regardless of whether there is a visible fossil nucleus or not. Furthermore, it is evident that cementation and even recrystallization can happen quickly if the conditions are right on timescales of several years (Fig. 8C) (*e.g*., Coleman, 1993; Melim and Spilde, 2011). Thus, unresolved outstanding issues about concretions include our ability to distinguish the biotic and abiotic processes involved in their formation, and the possibility that biomineralization can occur by microbial alteration of the pore fluid chemistry to a thermodynamically favorable environment that triggers precipitation.

Concretions are of great interest in planetary exploration, as “blueberries” believed to be concretions have already been found at multiple places on Mars (*e.g*., Chan *et al.*, 2004, 2005; Squyres *et al.*, 2004; Grotzinger *et al.*, 2005; Calvin *et al.*, 2008). Concretions are evidence of groundwater involved in cementation, and thus, if Earth examples preserve biosignatures, there is a similar possibility that such signatures could be found on other planetary bodies such as Mars.

##### 5.2.1.4. Micron-scale biosignatures

Microbial body fossils can persist over billions of years on Earth, for example, in units such as the 1.88 Ga Gunflint Chert (*e.g*., Barghoorn and Tyler, 1965; Schopf *et al.*, 2002). For rocks older than ∼2 Ga, there is morphologic evidence at macroscale (see section 5.2.1.2) and microscale, which, combined with petrographic data and chemical signatures (*e.g*., stable isotope data, mineral phases, kerogen), allows for unambiguous interpretation of these as biogenic features (*e.g*., Schopf *et al.*, 2002, 2007, 2018). Unfortunately, in most rocks of this age, these signatures are often ambiguous due to geologic processes that over time alter and obscure much of the original fabrics, such as original minerals or cellular remains, at the microscale. Pore spaces within rocks can preserve body fossils (Lanier, 1989) or other evidence of biotic interactions, but they can also be filled in with abiotic carbon-rich fluids (Bower *et al.*, 2016).

Archean cherts, especially, exhibit both biotic and abiotic features that can be morphologically and chemically similar at the micron scale (Bower *et al.*, 2016). Chemical gradients that record fluid/rock/biota interactions within diagenetic cements can be observed in thin section and can provide useful information, and this remains to be further developed as a tool for interpreting biogenicity (Potter-McIntyre *et al.*, 2014).

Biotic and abiotic jarosite [KFe(SO_4_)_2_(OH)_6_] is indistinguishable at micrometer to submicrometer scales. A similar conclusion regarding scale was reached following experiments in which biomediated and abiotic mineral precipitates of Ca-sulfates were compared: compositional differences were apparent only at the submicron scale (Bower *et al.*, 2015). This is also true for mineral habits: mineral examples created in the laboratory via biotic and abiotic processes often cannot be differentiated unless examined at the submicron scale. It is imperative to consider scale context when searching for biosignatures, and some biosignatures may need to be examined over multiple scales for unambiguous interpretation.

#### 5.2.2. Preservation potential of objects as biosignatures

Inorganic processes that affect sediments following deposition are broadly referred to as “diagenesis.” The field of “taphonomy” (Efremov, 1940) studies how the biological remains and/or the by-products of organisms are transformed and preserved as they pass from the biosphere to the lithosphere (Cadée, 1991; Behrensmeyer *et al.*, 2000; Allison and Bottjer, 2011). Both diagenesis and taphonomy affect the overall preservational potential of biosignatures.

There are positive factors that favor preservation. (1) Rapid and early diagenetic cementation in detrital systems that lowers sediment permeability, which along with anoxia, can greatly reduce rates of organic matter degradation and can essentially “freeze” biogenic features and help preserve them. (2) In chemical sedimentary systems, preservation is enhanced by rapid entombment in finely crystalline chemical precipitates, particularly where primary mineral phases are chemically stable and resist dissolution and aqueous weathering (Farmer, 1999b). On Earth, these favorable lithotypes include cherts and phosphorites, along with less stable carbonates and shales, which are the most common host rocks for the fossil record on Earth. (3) Selective biases such as mineralized skeletons (Dart, 1949; Lawrence, 1968; Olson, 1980; Seilacher, 1992) or large organisms and collections of organisms (biofilms, microbial mats) can also have higher preservation potential than individual microbes (Bower *et al.*, 2017; Hays *et al.*, 2017).

The potential for a preserved record of extraterrestrial microbial life on other planets in our Solar System, such as Mars (*e.g*., Farmer 1995; Farmer and Des Marais, 1999; Ruff and Farmer, 2016), has been fueled by studies of microbial biosignature preservation in a variety of modern and ancient terrestrial analog environments (*e.g*., Konhauser *et al.*, 2001; Schopf *et al.*, 2012). It is also important to understand the different environments and controls that exist and operate on planets. For example, a 3.5 Ga rock on Mars will not have undergone the extensive metamorphism of a similar age rock on Earth nor will it have been in contact with diagenetic fluids for billions of years. Even young Earth rocks (several millions of years) often show evidence of superimposed multiple precipitation/dissolution events and mobilization of diagenetic minerals (*e.g.*, Potter *et al.*, 2011).

Based on extensive studies on Earth, it is apparent that biomineralization and preservation of biosignatures are dependent on natural context. A holistic approach of the entire environment—from the kilometer down to the submicron scale—needs to be examined, as opposed to just the “parts.” Another helpful approach could be to develop probabilistic models to reduce uncertainty in biosignature confirmation (*e.g*., Bayesian statistics approach of Walker *et al.*, 2018). These models can be used together with more traditional physical data to build a more robust approach to biosignature identification to deal with degrees of certainty.

Not all fossils will likely be pristine or unambiguous, and this can be quantified by careful measurement of the number of individual organismal fossils, number of traits or characteristics, sizes, and population densities of organisms. This is already done in the paleontology community where face recognition-type algorithms have been used to automatically identify trilobites (*e.g*., Wei, 1994; Cope *et al.*, 2012).

### 5.3. Patterns

**FIG. 9.**
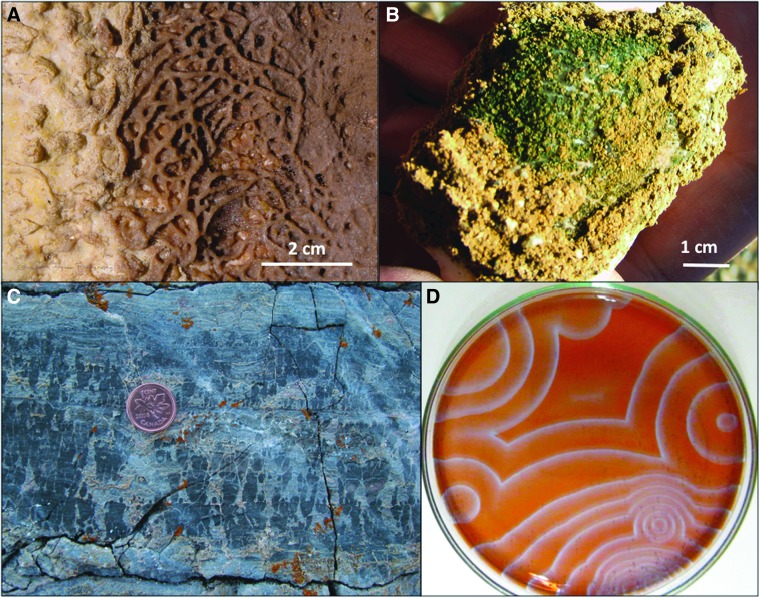
Mineral patterns can be biomediated **(A–C)** or a result of abiotic chemical reactions **(D)**. **(A)** A modern lithification front of biovermiculation, Cueva de Villa Luz, Mexico. View ∼8 cm across. Image: K. Ingham. **(B)** The underside of a hypolithic rock shows highly miniaturized biovermiculation patterns of cyanobacteria (genus *Chroococcidiopsis*) that live along the soil interface, Strzelecki Desert, Australia. Image: P.J.B. **(C)** Centimeter-size columnar-branching and multifurcate dolomitic stromatolites show millimeter-thick lamination patterns from the Paleoproterozoic McLeary Formation of the Belcher Supergroup, Canada. Image: D.P. **(D)** In an established abiotic B-Z reaction, chemical oscillation rings create life-like patterns. In this example, the red color arises from the redox indicator ferroin (commercial 25 mM phenanthroline ferrous sulfate) used in the experiment, and blue-gray lines represent redox fronts extending radially outward from oxidation spots in the geometric centers. Glass dish diameter 100 mm. B-Z, Belousov–Zhabotinsky. Image: D.P.

Here, we define a biopattern as a spatial and/or temporal organization of any of the substances and objects produced directly or indirectly by the processes of life discussed above, and a biosignature in its true sense. One of the earliest interpretations of biopatterns as biosignatures dates to Xenophanes (c. 570 BC–c. 475 BC), who observed structures in rocks inland from the ocean that resembled marine shells and fish and hence fossils (biosignatures) and concluded that an ocean (containing bivalves and fish) once occupied the inland region (Burnet, 1930; McKirahan, 1994). Of course, modern examination of fossils and their morphology and possibly chemical life traces are central to paleontology, biology, and geology and offer a record of evolution and the history of life. However, we now understand that there is a vast array of potential biopatterns ranging in scale from nanoscale biochemical patterns to multiple kilometer-scale brushlands and forest tree growth patterns. Such patterns do not rely on a particular chemistry or morphology, but only that patterns of some sort can be recognizable, analyzable, and ultimately tied to specific biological processes (Fig. 9A–C).

Abiotic reactions can create life-like patterns (Fig. 9D), but when clear biologically controlled patterns exist and abiotic mimics adequately ruled out, then they may be strong candidates for consideration as universal biosignatures (Schubert *et al.*, 2017). In this work, we focus only on physical manifestations of visually identifiable spatial patterns.

Physical patterns can vary widely in characteristics but are typically sinuous, curving, or spatially arranged due to the physics that govern biological growth (Meron *et al.*, 2004). Biopatterns can be influenced by environmental factors such as ultraviolet light, as is done in industrial biopatterning for biomedical applications from tissue engineering to fundamental cell studies (Whitesides *et al.*, 2001). Biopatterns can also be influenced by the presence or absence of sunlight. The shape of stony corals (hexacorals) changes as a function of water depth, which consequently affects light penetration. For example, branching in *Porites sillimaniani* decreases with depth (see Kaandorp and Kübler, 2001).

Biopatterns can include concretions (Suga and Nakahara, 2012; Yoshida *et al.*, 2015), layering in stromatolites (Semikhatov *et al.*, 1979; Awramik, 1992), and biovermiculations in caves and deserts (Thiéry *et al.*, 1995; Klausmeier, 1999; HilleRisLambers *et al.*, 2001; Schubert *et al.*, 2017). Biopatterns are not just passive responses of biological systems. For example, increasing density of soil crust patterns is correlated with diversity, metabolic activity, and capacity to restructure the soil (Mogul *et al.*, 2017).

Biovermiculations are worm-like or hieroglyphic-like patterns often occurring in biological mats or thin films formed by communities of microbes. Most commonly, biovermiculations (bioverms) occur in caves (Fig. 9A) or ancient ruins but can be found in desert soil crusts, hypersaline creek algae growth, and even in modern walls and buildings. Of particular interest in the study of early life and life in extreme environments is that microbial communities can grow under hypolithic rocks (Fig. 9B), providing a small “greenhouse”-like environment where biology can be protected in an otherwise uninhabitable or deadly environment.

The patterns can be traced back to the early studies in morphogenesis (*e.g*., of the coloration patterns on animals) (Turing, 1952). In resource-constrained environments, biological systems form patterns that may serve to optimize their return on the effort to acquire needed resources (Schubert *et al.*, 2017). These patterns persist over time, partly because cave environments are not perturbed by surface weather and only rarely affected by events such as flooding or animal activities. Thus, microbial activities result in ongoing mineralization of patterns that can provide evidence of life even when microbial activities may have long ceased.

Biopatterns can be preserved across geological timescales, but challenges to preservation (Hays *et al.*, 2017) exist, most notably the living structures must be covered rapidly or self-mineralize (*e.g*., Boston *et al.*, 2001). DNA and protein sequence information typically undergo rapid degradation over very short geological timescales, with the exact amount of time dependent on environmental factors such as temperature, humidity, and the encasing matrix. However, diagenesis can preserve biochemicals such as amino acids from structures such as eggshell and bone (Bada *et al.*, 1999), allowing inference of the original shape of soft-body parts.

In addition, characteristic laminated structures of stromatolites (Fig. 9C) are easily recognized over 3.5 billion years of the geologic record (Hofmann *et al.*, 1999; Allwood *et al.*, 2006), although many ancient putative examples still engender heated debate. Currently, a debate exists over the putative stromatolites from the 3.7 Ga Isua supercrustals (Nutman *et al.*, 2016; Allwood *et al.*, 2018). Stromatolites are still forming today in many different environments, including normal salinity marine, hypersaline marine, streams, lakes (both freshwater and saline), and thermal springs. Stromatolite structures are a primitive, fundamental outcome of life adapted to living in shallow photic environments, and their morphological preservation is rationalized to have been more favorable before the advent of organisms that graze on them. Biopatterns in caves commonly also lithify even as they grow, providing a mechanism for their long-term preservation (Boston *et al.*, 2009).

Biopatterns in surface environments must be entombed and permineralized like traditional fossils or encased in various salts and other evaporites for preservation. Even with all these challenges, on a planet where biology has spread globally and existed for billions of years (*e.g*., Earth and potentially early Mars), it is reasonable that a large number of biopatterns would be preserved and could thus be interpretable as biosignatures.

#### 5.3.1. Biopatterns in stromatolites

Stromatolites and other microbialites constitute an important group of biosignatures that present biopatterns (Fig. 9C). The term microbialite (Burne and Moore, 1987) encompasses various types of organosedimentary deposits that bind and trap sediment, including stromatolites (laminated), thrombolites (clotted), dendrolites (composed of cm-size shrubs), and leiolites (which are structureless). These four microbial types have biopatterns, but establishing the role, if any, of biology in forming the structures or patterns has been contentious. Stromatolites have been at the forefront of this debate (Grotzinger and Knoll, 1999; Awramik and Grey, 2005; McLoughlin *et al.*, 2008; Allwood, 2016).

Numerous criteria have been developed to increase the level of confidence that a stromatolite is biogenic (Awramik and Grey, 2005). Given these, biopatterns occur at three different observational levels: macrostructure, mesostructure, and microstructure. Macrostructure refers to the overall shape. Common shapes include millimeter- to decimeter-size distinctive cones, domes, and columns. Some shapes are difficult to attribute to nonbiologic processes, specifically when their geologic context is considered (*e.g*., in subaqueously deposited sedimentary rocks, primarily carbonates). Mesoscale is intermediate between macro- and microstructure and refers to the internal structure visible to the unaided eye and serves as the scale for identifying the four types of microbialites. The defining characteristic of a stromatolite is lamination. A common biopattern is alternating, thinner dark laminae with thicker light laminae (at the millimeter or less scale), with laminae across the structure having variable thicknesses (non-isopachous). Microscale structure is studied with the aid of a microscope. Biopatterns include the growth position of microbial fossils, the arrangement of sediment grains, and the sharpness of the boundaries between dark and light laminae.

Microorganisms living in chemical sedimentary accreting systems influence the form of precipitation indirectly by providing surfaces for mineral nucleation. These influences and inherited forms are difficult to distinguish at the nanoscale but are often seen clearly at larger spatial scales. Visible laminae such as those used to define true stromatolites (vs. abiotic examples such as the lamina inside a geode) are usually visualized and studied at spatial scales much coarser than unit cells, although the cells provide the basic building blocks.

In biofilms, organic forms are mostly visualized at the micron scale, where they provide templates for mineral precipitates that nucleate on them and eventually entomb whole organisms. The form of a captured organism may retain important aspects of the external shape of cells during accretion. However, only the basic aspects of the inherited form are usually preserved, and, once the organism is fully entombed, it no longer controls the inherited form, because the geometry of unit cell accretion is controlled inorganically at a fine scale.

Although stromatolites form only rarely in surface environments today (McNamara and Awramik, 1992), they are relatively common in the fossil record, particularly in the Proterozoic (Peters *et al.*, 2017). Stromatolites are readily seen by the naked eye and criteria have been developed to establish their biogenicity. In many caves, stromatolite-like laminated structures are very common and formed microbially but not in response to light direction as they are not photosynthetic but heterotrophic or chemolithotrophic (Melim *et al.*, 2009). They usually are pendant structures that grow in pools and fossilize very well, but living examples are known (Melim *et al.*, 2015).

Computational modeling offers a novel way to distinguish biological from abiological patterns. If the “ruleset” of the generating system (*e.g*., the environmental factors and resulting organism behaviors that lead to the pattern) can be identified, it can then be compared with known biological and abiological systems. Differential equation-based models (von Hardenberg *et al.*, 2001; Meron *et al.*, 2004) and cellular automata (Dunkerley, 1997; Schubert *et al.*, 2017) are equivalent ways to model biological systems *in silico* (Strader *et al.*, 2011). Determining mathematical and statistical rules that govern biological growth processes and their interactions with mineralization processes is nontrivial but important for many problems. Consequently, various techniques have been used, including machine learning (Richards *et al.*, 1990; Campbell *et al.*, 2004; Placzek, 2014; Gurikov *et al.*, 2016), coevolution (Juille and Pollack, 1998), and histogram-based methods (Schubert *et al.*, 2017).

One method currently being explored that directly observes pattern formation involves taking time-lapse images of slowly growing biological communities that develop recognizable patterns such as biovermiculations or mat structures. By comparing two successive pictures, one can see how they have changed spatiotemporally, which in turn specifies the rules to be used in the modeling. Time series comparison is straightforward and thus the basis of most techniques. In contrast, only one high-resolution picture is needed using histogram techniques (Schubert *et al.*, 2017), because only spatial comparisons are done, making it a particularly promising method for examining rock strata or other potentially biological patterns.

**FIG. 10.**
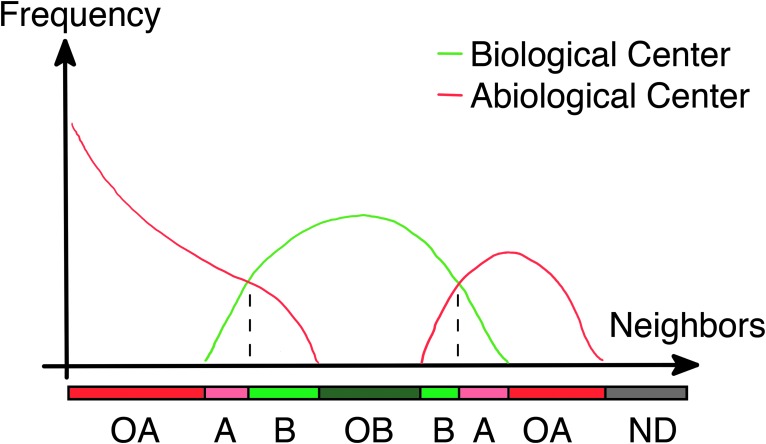
Histogram comparison used to determine a ruleset for a biopattern. Neighbors are the number of cells with biology within a preselected range, called the radius. Frequency is the number of cells (either abiological or biological) in the entire image that have the number of neighbors specified by the horizontal axis. The image is not guaranteed to have all possible configurations of neighbors, so there are sometimes no data available. These patterns specify the underlying rules. A, abiological; B, biological; ND, no data; OA, only abiological; OB, only biological. Image: K.E.S.

In the histogram method (Fig. 10), any region of interest in a biopattern image is first selected and converted to two colors (*e.g*., Red = R and Green = G) based on clustering or user experience. Separate histograms are then made around each R or G pixel, indexed by the number of neighbors that are green in a preselected region around the points, designating these as histogram R and histogram G. The number of neighbors corresponds to the density of green pixels in that region, which roughly corresponds to the amount of biological competition for some limiting factor. The histograms are then compared with each other bin by bin, where either histogram R or G will have a higher frequency, that is, the density of red or green pixels at a given distance from a red or green pixel is determined by the ruleset, which becomes evident in the histogram. Such a comparison is shown in Fig. 10, in which red corresponds to abiological material and green corresponds to biological material.

**FIG. 11.**
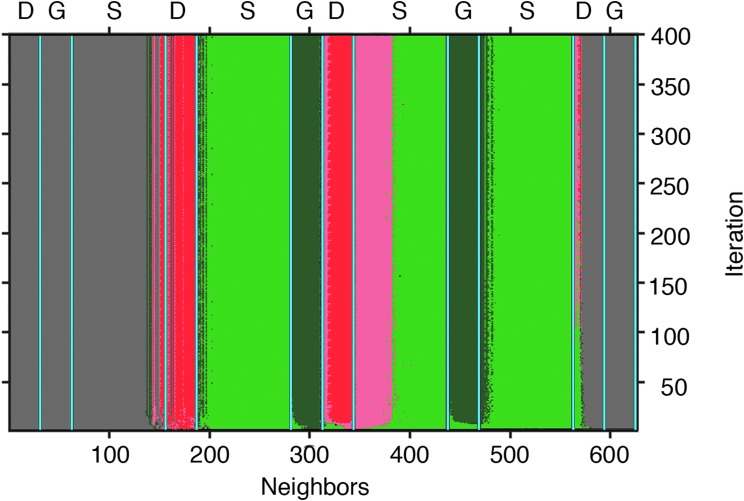
Histogram comparison of the number of neighbors for biological and abiological “cells” at each iteration of a modeled cellular automaton versus a ruleset determined from an actual biological system. The vertical axis is iterations (essentially time). Each row is a histogram of the cellular automaton state, where color indicates the relative amount of biological versus abiological “cells.” Gray indicates no data, green indicates more biological “cells” at that number of neighbors, while pink indicates more abiological. Dark green indicates only biological cells having that number of neighbors and dark pink indicates only abiological cells having that number of neighbors. Image: K.E.S.

The histogram method has been validated using single states of a known cellular automaton, and the predicted ruleset compared with an actual ruleset. The result for one such system is shown in Fig. 11, where the ruleset was estimated at each iteration of the cellular automaton only using the histograms from that iteration.

The actual rule boundaries are shown by the light blue lines and the rules are listed at the top. Gray indicates regions lacking data (*i.e*., where no cell had that minimum number of neighbors). Dark green indicates that only histogram G (biological) had a nonzero value for that density/neighbor count, while dark pink indicates only histogram R (abiological) had a nonzero value. Light green indicates that histogram B had a higher frequency than histogram A at that density, and light pink indicates that histogram A had a higher frequency than histogram B at that density. It is readily apparent that boundaries between the histogram regions outlined above correspond very closely to the actual rules for almost all iterations after an initial start-up. Although the spatial pattern of pixels changes continuously over time according to the ruleset, the relative frequencies stay constant, because these are defined by the rules. Assuming that a pattern encountered was well established, the histogram technique should give a good estimate of the rules, which can then be used to putatively distinguish biological from abiological patterns.

While more work needs to be done to develop these histogram techniques, biopatterns offer an exciting potential biomarker that could be detected by using machine-learning techniques. Machine-learning schemes that can use such cellular automata logic and can build up a basis of experience over the course of training can function much like a well-seasoned field scientist but with the added benefit of immediate quantitative rationales for making a particular biotic/abiotic call on a specific pattern. The application of such automated decision calls on patterns can be applied to future robotic missions to planetary targets of astrobiological interest to provide immediacy of remote decision-making without continuous direct Earth control.

##### 5.3.1.1. Abiotic chemically induced patterns

Many geological processes are driven by cyclic processes, including seasons, tides, and day/night cycles. In turn, these large-scale physical processes can induce cyclic behaviors in biological and geochemical systems. Indeed, stromatolite laminations have been attributed in some cases to seasonal variation and day/night cycles among other forcing factors (Seong-Joo *et al.*, 2000). There are also a variety of regular banding patterns observable in many mineral types, for example, the banding observed in minerals such as malachite [Cu_2_CO_3_(OH)_2_]. A possible explanation for some of these regular mineralogical patterns has been attributed to Liesegang phenomena, which are the result of reaction/diffusion-type physicochemical systems (Hartman *et al.*, 1934). A variety of such systems have been studied in the laboratory, and collectively the ability of both natural biological and abiological phenomena to produce intriguingly complex patterns is well known (Ball, 1999) (Fig. 9D).

The potential importance of reaction diffusion systems in biosignature detection is that there may be a variety of phenomena that can produce seemingly highly ordered systems, which may not be easily attributable to or distinguishable from biological processes. Besides those already identified, there may be many that remain to be discovered and that may operate over much longer timescales.

#### 5.3.2. Isotopic patterns

Physical, chemical, and biological processes can all produce stable isotope fractionation. Fractionation of isotopes among states, phases, and biological organic matter is driven by differences in the energy stored in bonds. Thus, patterns of isotopic abundances between substances can reflect these differences, but their combination with contextual data substantially strengthens the veracity of isotopic patterns as biosignatures. Two major elemental systems are described below.

##### 5.3.2.1. Carbon isotopes

One example of a biological isotope fractionation process is the fixation of inorganic carbon by autotrophs. In these pathways, CO_2_ containing the lighter isotope of carbon, ^12^C, is energetically more easily manipulated than CO_2_ containing ^13^C, resulting in enrichment in ^12^C in the resulting fixed organic carbon. The utility of stable isotope fractionation patterns as a biosignature depends on the isotopic composition of both the putative biological material and its starting material. However, some abiotic chemical processes produce isotopic fractionations similar with biological ones and biogenicity is often hotly debated on both sides, for example, see the disagreement over interpretation of iron isotopes in the ancient rock record in Guildbaud *et al.* (2011) and Czaja *et al.* (2012). Environmental context supports the observed fractionation as abiotic if mineralogical and geochemical processes alone can explain the observed signatures. Alternatively, the presence of complex organic compounds and metabolic products might suggest biological element cycling with isotopic fractionation. Patterns of isotopic abundances, among individual types or classes of biomolecules, can be very compelling as biosignatures (*e.g*., Hayes, 2001).

##### 5.3.2.2. Sulfate isotope signatures

Transition from abiotic to biotic: In early Earth history before the rise of abundant atmospheric O_2_, abiotic photochemistry of S-species derived from volcanic outgassing was a primary driver for cycling surface sulfur. These gas-phase photochemical reactions produce mass-independent sulfur isotope fractionation that can be detected in sulfides older than 2.4 Ga. There is evidence for microbially mediated sulfur and sulfate reduction in the Paleoarchean (Shen *et al.*, 2001; Philippot *et al.*, 2007; Wacey *et al.*, 2010). As atmospheric O_2_ levels increased during the early Paleoproterozoic due to the rise of oxygenic photosynthesis, evaporitic sulfates accumulated in the sedimentary rock record, along with isotopic evidence for further biologically mediated sulfate reduction beginning around 2.7–2.5 Ga (Canfield and Raiswell, 1999).

The effects of biology on S cycling are also intertwined with other direct and indirect biological processes. Most notably, the rise of biogenic atmospheric O_2_ significantly altered the photochemical processes that affect S exhaled by volcanism, and this has left a strong isotopic fractionation signal in the geological record (Farquhar *et al.*, 2000). By the time of the GOE, biology and oxidative weathering, both of which exhibit mass-dependent fractionation, had come to dominate S cycling.

Uncertainties persist when isotopes are invoked as a single line of evidence for a biosignature, especially for samples from the early Archean (3.7–3.8 Ga). For example, δ^13^C values of graphite in a highly metamorphosed sedimentary rock are not a reliable indicator of biogenicity because there are nonbiological processes that can produce graphite having δ^13^C values that are more negative, relative to coexisting carbonates, similar to the pattern that is observed for organic products of biological metabolism (*e.g*., Ray, 2009). Hence, the δ^13^C of graphite should be regarded as a consistent but insufficient criterion for biogenicity. This criterion partially explains continuing controversies about the earliest Archean biosignatures. However, in the case of younger (3.47 Ga) less metamorphosed rocks with carbon (particulate kerogen), isotopic biosignatures are less controversial (Schopf *et al.*, 2018).

## 6. Summary and Recommendations

The concept of “biosignatures” encompasses a suite of continuous phenomena with end members of life versus non-life for different features and parameters, many of which can act synergistically or antithetically with each other producing a great deal of complexity. All interpretations are further complicated by the fact that there are many overlaps between biotic and abiotic signals in the rock record when studies focus on individual signatures or even just a few signatures taken together. There is the additional complication that biotic versus abiotic features may appear very similar or even identical under some circumstances and may require several lines of evidence to determine their true origins. The field of astrobiology and the search for extraterrestrial life are heavily biased by our singular terrestrial perspective. Current knowledge of biosignatures is fundamentally based on three classifications of expressions of life: substances, objects, and patterns, but there may be additional dimensions that require consideration. Given these daunting challenges for biosignature identification, we discuss three approaches for future research in astrobiology.

### 6.1. Scales and context

There is a fundamental need to understand biosignatures in their geologic context and across multiple spatial and temporal scales. It is important to look at the whole picture, not just the individual part or sum of parts of a system. Spatial scaling and the distribution relationships can determine the mappable extent of biosignatures. There is a tendency in science to focus examination of biosignatures at one particular scale, particularly when searching for an analogue to a specific terrestrial example. However, the complexity of biosignatures requires an integrative approach encompassing tools and methodologies as well as the context for each scale and type of data. Both classical and newer analytical methods will need to be used, as well as approaches that can span traditional research boundaries.

Astrobiology as a discipline needs more analog studies at multiple nested scales, with clear context for each example. Spatial and temporal scales (both modern and ancient) are also important. For the best understanding of how biosignatures form and are preserved, we must account for macroscale characteristics all the way down to sub-microscale ones, with lateral as well as vertical distribution and characterization.

Future studies should clearly establish the context and conduct examinations across multiple scales, wherever possible. Potential biosignatures can be ambiguous yet intellectually seductive, and thus, many independent lines of evidence and tools are required to keep us honest in our inquiries (Boston *et al.*, 2001). Quantification of biosignature metrics whenever at all possible can help establish detection confidence and allow for more confident assignment of “weightings” or ranked prioritizations that can be used in the search for life.

### 6.2. Community-accepted standards

How can one determine whether a planetary phenomenon is the result of life? A set of community-accepted standards of abiological versus biological characteristics is needed. There is likely little left in Earth's near-surface and subsurface environment that has not been altered in one way or another by biological processes, and thus, we may need to rely heavily on controlled abiotic experiments to provide “clean” determinations. A set of standards to be used globally across multiple instrument platforms would be helpful.

Simulation of abiotic conditions in controlled experiments is one way to attempt to define the abiotic “end member.” Samples containing known biosignatures can be experimentally altered and aged (*e.g*., by placing them under temperature and pressure conditions simulating diagenesis). It remains unknown whether such experiments can be scaled directly to natural systems. For example, it is notoriously difficult to reproduce the processes of petroleum and coal formation in the laboratory; these processes appear to require long periods of heating at relatively low temperatures. It is not always possible to substitute higher intensities of temperature or pressure for time, and there may be many unknown feedbacks occurring in natural systems or even understood feedbacks that cannot be produced in the laboratory.

More community-accepted standards of abiosignatures or abiotic features would be tremendously helpful, just as analytical standards can be critical to provide consistency in measurements across multiple instrument platforms. Such standards are still being developed and debated by scientists. The development and use of mathematical models and statistical methods to examine probabilities for accurately identifying biotic processes over abiotic ones may have application at multiple scales. Such mathematical models and patterns could also be used together with traditional, physical data for a more robust approach to biosignature identification.

### 6.3. Data management

The power of cybertechnology offers opportunities at every stage and level of the study of astrobiology, the search for biosignatures, and the attempts to understand the origins of life. A universal data management system could handle appropriate curation and cross-calibration of standards. More open data and sample sharing would facilitate interdisciplinary and international collaboration and the use of data analytics to identify the best pathways forward.

Astrobiology research can be high risk because there are so many overlapping variables to consider, because there is often such lack of consensus as to what constitutes a biosignature, and because it requires so many different disciplinary approaches to effectively answer the outstanding questions. However, more interdisciplinary collaboration among scientists studying astrobiology, and support for open data sharing and data management systems, can be effective in bridging communication and overcoming disciplinary barriers, transforming the science and the potential for new discoveries across multiple temporal and spatial scales (*e.g*., Park Boush *et al.*, 2017). Although the infrastructure to accomplish the goals that we set forth here may require considerable investment of effort and funding, it could provide multiple benefits for the astrobiology community, including access to user-friendly tools for data mining of existing data sets that would also be engaging to citizen scientists, students, and educators.

Coupled with the way science is conducted, there are additional challenges with biosignatures that are physical objects, namely their curation, an area that seems to have fallen out of favor in modern times but which is critical to our knowledge base. Importantly, such physical collections must be collated with all their associated information (*e.g*., metadata). A data management system for biosignature and abiosignature samples would further facilitate interdisciplinary collaboration in that working on and thinking about a common body of samples could bring great intellectual power to bear on the challenges articulated here. Other paleobiology databases (*e.g*., paleobiodb.org) and stratigraphic databases (*e.g*., macrostrat.org) are helping geoscientists sort out important temporal and spatial relationships in searchable, aggregator platforms.

Data management systems would facilitate new exploration by using “big data,” especially for a field such as astrobiology that relies on interdisciplinary data. Complex patterns and relationships could be discoverable by leveraging the cyber infrastructure and technology currently available but underutilized. The network of relationships between minerals and biology (Hazen *et al.*, 2008) is an example of a major discovery facilitated by computational collaborations.

An integrated GIS (Geographic Information System) framework of databases that can be interrogated with a search engine, or having multiple layers of science information, including spatial locations, is a long way off. However, the vision of how it can benefit the community has to start now, even though this is a long-term investment.

In the cosmic perspective, terrestrial bias still makes it difficult to fully understand what an abiotic, habitable planet would look like. Yet at the same time, we are at an exciting cusp, poised to move forward with an enthusiastic and interdisciplinary community of scientists and an expanding toolbox of methods to uncover more details about both biosignatures and abiosignatures. An integration of studies across scales and contexts, using quantitative methods involving agreed-upon standards, and making use of cyberinfrastructure, will collectively guide future explorations for the origins of Earth life and the potential existence of biosignatures in extraterrestrial examples.

## Notes

Associate Editor: Lewis Dartnell

## Abbreviations Used

ELSI: Earth-Life Science Institute
EON: ELSI Origins Network
FTT: Fischer–Tropsch Type
GIS: Geographic Information System
GOE: Great Oxidation Event
LNRE: Large Number of Rare Events
MISS: microbially induced sedimentary structures
ROS: reactive oxygen species
SEM: scanning electron microscopy
